# Trilobatin ameliorates insulin resistance through IRS-AKT-GLUT4 signaling pathway in C2C12 myotubes and ob/ob mice

**DOI:** 10.1186/s13020-020-00390-2

**Published:** 2020-10-12

**Authors:** Min Liu, Lujing Wang, Xigan Li, Yucui Wu, Fei Yin, Jianhui Liu

**Affiliations:** 1 Chongqing Key Lab of Medicinal Chemistry & Molecular Pharmacology, Chongqing University of Technology, Hongguang Road 69, Ba’nan District, Chongqing, 400054 China; 2 College of Pharmacy and Bioengineering, Chongqing University of Technology, Chongqing, 400054 China

**Keywords:** Glucose transporter 4 (GLUT4), Insulin receptor substrate 1 (IRS1), Insulin resistance, Protein kinase B (PKB/AKT), Trilobatin

## Abstract

**Background:**

Trilobatin, a natural compound, has been found to exhibit anti-diabetic properties in high-fat diet (HFD) and streptozotocin (STZ) induced type 2 diabetic mice. But up to now no research has been reported on the effect of trilobatin on insulin resistance in peripheral tissues. Herein, we determined the effects of trilobatin on insulin resistance in palmitate-treated C2C12 myotubes and ob/ob mice.

**Methods:**

Male ob/ob mice (8-10 weeks) and same background C57BL/6 mice were used to evaluate the role of trilobatin on insulin resistance; protein expression and phosphorylation were measured by western blot; glucose uptake was determined a fluorescent test.

**Results:**

Treatment with trilobatin prevented palmitate-induced insulin resistance by enhancing glucose uptake and the phosphorylation of insulin resistance substrate 1 (IRS1) and protein Kinase B, (PKB/AKT), recovered the translocation of GLUT4 from cytoplasm to membrane, but preincubation with LY294002, an inhibitor of PI3K, blocked the effects of trilobatin on glucose uptake and the distribution of GLUT4 in C2C12 myotubes. Furthermore, administration with trilobatin for 4 weeks significantly improved insulin resistance by decreasing fasting blood glucose and insulin in serum, enhancing the phosphorylation of IRS1 and AKT, and recovering the expression and translocation of GLUT4 in ob/ob mice.

**Conclusions:**

IRS-AKT-GLUT4 signaling pathway might be involved in trilobatin ameliorating insulin resistance in skeletal muscle of obese animal models.

## Highlights

- Trilobatin improves insulin resistance in skeletal muscle.
- Trilobatin enhances glucose uptake in insulin-resistant C2C12 myotubes.
- IRS-AKT-GLUT4 signaling pathway is involved in trilobatin improving insulin resistance.

## Background

Increasing evidence indicates that obesity and type 2 diabetes has elevated over years in both developing and developed country, which are closely associated with insulin resistance, a pathophysiological condition characterized by an impaired insulin action in insulin-sensitizing tissues including liver, adipose tissue and skeletal muscle [1, 2]. Skeletal muscle accounts for 40% of body weight in humans, and is responsible for up to 80% insulin-mediated glucose disposal under normal physiological conditions [3, 4]. To date, the role of skeletal muscle in glucose homeostasis has been widely reported in insulin resistance in both cellular and animal disease models [5–7]. However, although an enormous amount of mechanisms of insulin resistance has been postulated in skeletal muscle, the promising compounds are still needed to be explored.

Trilobatin, isolated from the leaves of *Lithocarpus polystachyus* Rehd, is a natural sweetener [8]. It has been shown that trilobatin has anti-oxidative [9, 10], anti-viral and anti-inflammatory activities [11, 12]. Trilobatin also exhibits anti-hyperglycemic properties by accelerating liver glycogen synthesis, decreasing oxidative stress, increasing the expression of glucokinase, and up-regulating the expression of insulin receptor substrate (IRS) on long-term double high-fat diet and streptozotocin (STZ) induced type 2 diabetic mice [13]. Similar to other dihydrochalcones, phlorizin (a dual SGLT1/SGLT2 inhibitor) [14], phloretin (a GLUT2 inhibitor) [15], and acarbose (an alpha-glucosidase inhibitor) [16, 17], our previous works demonstrated that trilobatin could be bind with SGLT1/2 [18]. But up to now, the effect of trilobatin on insulin resistance is still needed to be identified.

In the present study, the effects of trilobatin on glucose uptake and insulin resistance and the underlying mechanisms were investigated in palmitate-treated C2C12 myotubes and ob/ob mice, especially for the regulation of trilobatin on the phosphorylation of insulin receptor substrate (IRS) and PI3K/AKT, and the expression and translocation of glucose transporter 4 (GLUT4) in cellular and animal disease models of insulin resistance.

## Methods

### Materials

Trilobatin, palmitate, DAPI and (2-(*N*-(7-nitrobenz-2-oxa-1,3-diazol-4-yl) amino)-2-deoxyglucose (2NBDG) uptake measurement kits were purchased from Sigma (St. Louis, MO, USA). IRS1, p-IRS1 (Ser 612), p-IRS1 (Ser 307), Akt, p-Akt (Ser 473), p-Akt (thr308), Na, K-ATPase, MYH1, MYOD1, and β-actin primary antibodies were bought from Cell Signaling Technology (Danvers, MA, USA). GLUT4 antibody was obtained from Abcam (Cambridge, MA, USA). HRP-conjugated GAPDH primary antibody was purchased from Aksmics (shanghai, China), Specific anti-mouse and anti-rabbit HRP-conjugated second antibodies were obtained from Santa Cruz Biotechnology (Texas, CA, USA). Rat/mouse insulin ELISA kits (EZRMI-13K) and ECL chemiluminescence detection reagent were obtained from Millipore (Billerica, MA, USA). Plasma membrane protein extraction kit, nuclear/cytosolic fractionation kit, RIPA buffer and BCA protein assay kit and other chemicals were purchased from Beyotime (Shanghai, China).

### Cell culture

Mouse skeletal muscle cell lines, C2C12 myoblasts, were obtained from American Type Culture Collection (ATCC, Manassas, VA, USA) and maintained in DMEM supplemented with 10% FBS, and 1% penicillin/streptomycin (P/S) at 37 °C in a humidified incubator with 5% CO_2_ atmosphere.

The differentiated C2C12 myotubes were induced as descirbed before [19]. Generally, to induce the development of myotubes in C2C12 cells, the media was changed with differentiated media, which were prepared with fresh DMEM supplemented with 2% horse serum and 1% P/S, and continued to incubate for 4 days. After that, the cell morphology was observed and marker proteins (MYH1 and MyoD1) were determined by western blot. To induce the insulin resistance in differentiated C2C12 myotubes, the cells were starved in serum-free DMEM for 4 h, and then incubated with 0.2 mM PA for 24 h.

### Animals

Male ob/ob mice (8–10 weeks) and same background C57BL/6 mice were purchased from Tengxin Biotechnology Co. (Chongqing, China), which were allowed ad libitum access to food and water unless otherwise stated, and rooms were maintained at 22 °C and 50% humidity on a 12-h light/dark cycle. The mice were administrated with 10 mg/kg trilobatin (intragastric, i.g.) once for each day for continued 4 weeks, and the control mice were treated with same volume of in phosphate-buffered saline (PBS). Before dissection, the mice were euthanatized by CO_2_ inhalation, and gastrocnemius (the fast-glycolytic) muscles were isolated, froze in liquid nitrogen, and stored at − 80 °C. All animals’ experiments were carried out in accordance with the principles and guidelines of the Chinese Council Animal Care and also approved by the Institutional Animal Care and Use Committee at Chongqing Science and Technology Committee.

### Glucose tolerance test

Glucose tolerance test (GTT) was performed as previously described [20]. After the ob/ob mice were treated with 10 mg/kg trilobatin (i. g.) once for each day for continued 4 weeks, the mice were fasted overnight (12 h), 2 g/kg glucose was administrated intragastrically, blood samples were collected from tail vein at 0, 15, 30, 60 and 120 min respectively, and the blood glucose was determined with One Touch Ultra Mini Blood Glucose Monitoring System (Johnson, Life Scan, Inc., Milpitas, CA, USA).

### Palmitate solution preparation

BSA-bound palmitate was prepared according to the procedure described previously [21]. Palmitate was firstly dissolved in 0.1 M NaOH to a concentration of 75 mM by heating at 70 °C in a shaking water bath, and then the stock solution was complexed with 5% fatty acid-free bovine serum albumins (BSA; Ameresco, Solon, Ohio, USA) in PBS at a ratio of 8:1 (v/v) at 56 °C in a shaking water bath. After filtration (0.2 μm membrane filter), this solution was stored at − 20 °C and used within 2 weeks. The same concentration of NaOH mixed with 10% FFA-free BSA was used as a control.

### Glucose uptake

Glucose uptake in differentiated C2C12 myotubes was measured by adding 2-NBDG, a fluorescent d-glucose analog to trace the uptake of glucose directly [22, 23]. Generally, after the differentiated C2C12 myotubes were starved in serum-free DMEM for 4 h, and then treated with 0.2 mM palmitate in the presence or absence of indicated concentrations of trilobatin for 24 h, the cells were starved in KRBH for 3 h, and then incubated without or with 100 nM insulin for 30 min, after that, 2-NBDG with a final concentration as 100 μM was added and continued to incubate for 30 min. After incubation, free 2-NBDG was washed out 3 times with PBS, and fluorescence densities in cell monolayers were measured with a fluorescence microplate reader (TECAN, Swiss) at an excitation wavelength of 475 nm and an emission wavelength of 550 nm. The protein concentration of each sample was determined by the BCA method. Results were normalized by mg of total protein.

### Protein extraction

After washed once with ice-cold PBS, the skeletal muscle tissues or cells were homogenized with hand held homogenizer (Biospec, Bartlesville, OK, USA) for 10 s and incubated on ice for 40–60 min in a modified RIPA buffer in the presence of 1% protease/phosphatase inhibitor cocktail, and the protein concentrations were measured with BCA protein assay kit from Beyotime (Shanghai, China).

To prepare the membrane and cytoplasm proteins, after the gastrocnemius muscles or cells were washed with PBS, the proteins were obtained by using plasma membrane protein extraction kit and nuclear/cytosolic fractionation kit from Beyotime (Shanghai, China) respectively according to the suggestions from the supplier. Generally, after the gastrocnemius muscles (which were cut into small pieces before use) or cells were incubated with membrane protein extraction reagent A in the ice bath for 10–15 min, and homogenized 30–50 times, the lysates were centrifuged for 10 min at 700× *g* and at 4 °C to remove the nucleus and unfragmented cells. The cell membrane fragments were precipitated by centrifugation at 14,000× *g* and at 4 °C for 30 min, and the supernatant is absorbed as cytoplasmic protein. And then, the precipitate was adding 200 μL membrane protein extraction reagent B and was re-suspended at maximum speed of Vortex for 10 s, followed by an ice bath for 15 min. The lysates were centrifuged at 14,000× *g* and at 4 °C for 5 min, the supernatants were collected and used as the membrane proteins. After the proteins were quantified with BCA protein assay kit from Beyotime (Shanghai, China). Western blot was performed to determine GLUT4 expression on the plasma membrane and cytoplasm. The membrane marker Na, K-ATPase was used as a control in this study.

### Immunohistochemistry

After the mice were administrated with 10 mg/kg trilobatin for 4 weeks (once for each day), the skeletal muscle tissues were isolated and fixed (10% formalin solution in 0.1 M PBS), frozen at − 80 °C overnight, and cut into 10 μm sections on a freezing microtome (Leica, Nussloch, Germany). The sections were permeabilized in 0.1% Triton X-100 in PBS and blocked in 1% BSA in PBS before incubation with the primary antibody. After stained with GLUT4 antibody (ab654, Abcam Inc. Cambridge, MA, USA) and together with DAPI nuclear stain (Invitrogen, CA, USA). Fluorescence images were acquired using a confocal microscope (Nikon, Tokio, Japan).

### Western blot

Proteins (20–30 μg) were subjected to 10% SDS-PAGE and then transferred to a PVDF membrane (Immobilon P; Millipore, MA, USA), and the membranes were then blocked for 2 h at room temperature with 5% BSA in TBST (20 mM Tris, 150 mM NaCl, 2.7 mM KCl, 0.1% Tween 20, pH 7.4). The membranes were next immunoblotted with primary antibodies at dilutions of 1:500 to 1:2000. Specific total or phospho-proteins were visualized after subsequent incubation with a 1:10,000 dilution of anti-mouse or rabbit IgG conjugated to horseradish peroxidase. Excess antibody was washed off with TBST, and immunoreactivity was detected using ECL western blotting reagent (Millipore, MA, USA). Signal bands were quantified by densitometric analysis using ImageJ software (available from NIH at https://imagej.nih.gov/ij/) after scanning the blotted membrane. Three independent experiments were performed for each condition.

### Statistical analysis

The data are expressed as means ± SD. Statistical comparisons between two groups were carried out using Student’s *t*-test or one-way analysis of variance (ANOVA), and one-way ANOVA with Bonferroni post-hoc test for multiple comparisons. The differences were considered significant when *p* < 0.05.

## Results

### Trilobatin prevented the palmitate-induced decrease of glucose uptake in C2C12 myotubes

It has been established that the development of myoblasts is achieved with the expression of some myoblast-specific transcription factors, including myogenic determination protein 1 (MYOD1), myogenic factor 5 (MYF5), myogenin (MYOG) and myogenic factor 6 (MRF6) and some structural and enzymatic muscle-specific proteins such as myosin heavy chain 1 (MYH1), the main motor protein in muscle filaments [24]. In this study, C2C12 myoblasts were firstly incubated with differentiated media (DMEM supplied with 2% horse serum and 1% P/S) to induce the development of myotubes as described in “Methods”, the results demonstrated that the myoblasts were induced from human fibroblasts, and the myotube-like muti-nuclear structure were developed, which were confirmed by observing the morphology (Additional file 1: Figure S1A), detecting the protein markers MYH1 (Additional file 1: Figure S1B) and MYOD1 (Additional file 1: Figure S1C) of myotubes with western blot assay.

**Fig. 1:**
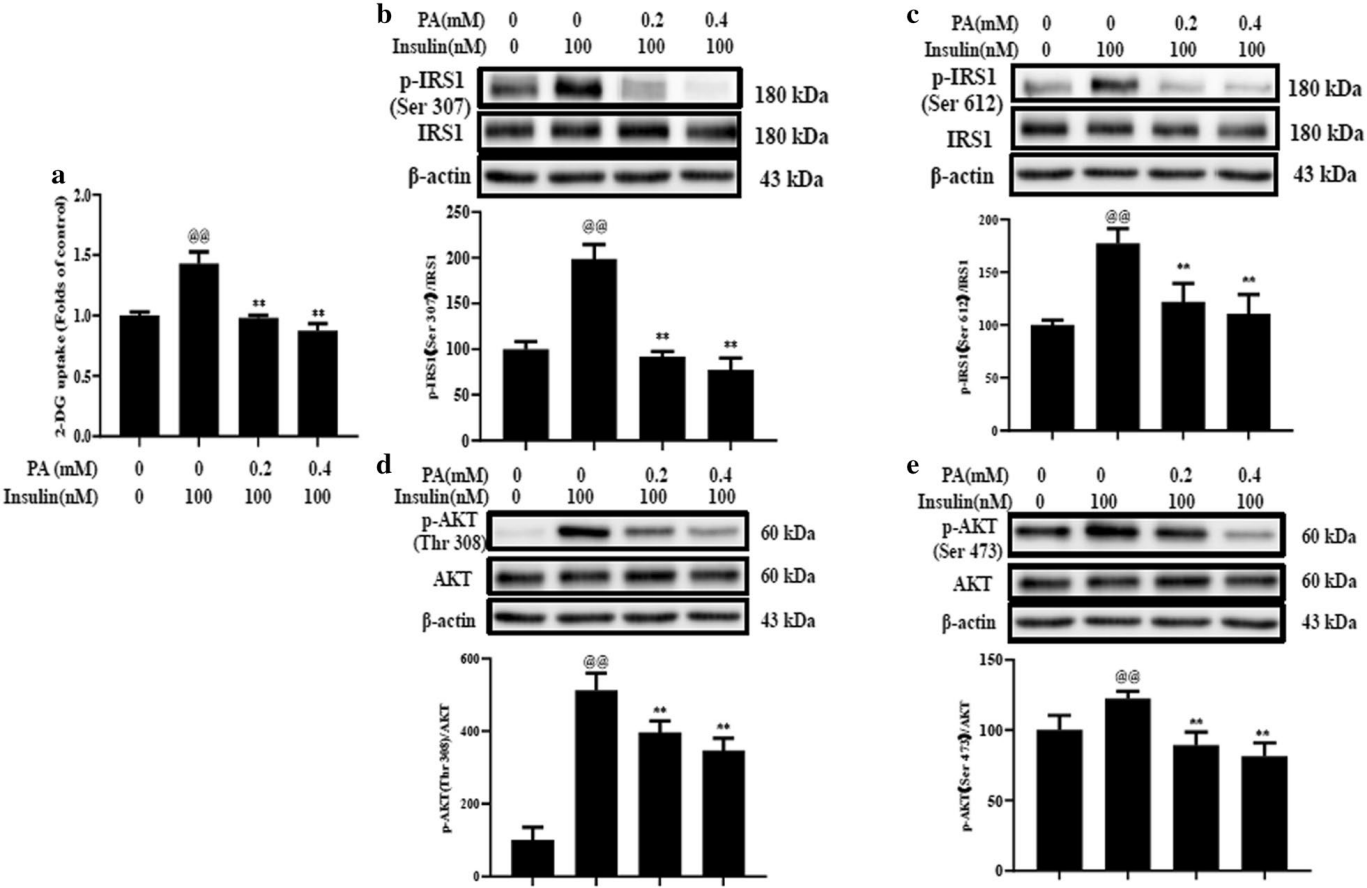
Palmitate induced insulin resistance in C2C12 myotubes. After the cells were starved for 4 h and incubated with 0.2 or 0.4 mM palmitate for 24 h. The glucose uptake (**a**) was determined with 2-NBDG kits, and the phosphorylation of IRS-1 at Ser 307 (**b**), Ser 612 (**c**), and AKT at Thr 308 (**d**) and Ser 473 (**e**) was detected with western blot. Data are mean ± SD (n = 3) from three independent experiments. ^@@^p < 0.01 vs. control (no insulin stimulation), **p < 0.01 vs. 100 nM insulin alone

To verify the effect of trilobatin on insulin resistance, we reproduced a classic cellular model of insulin resistance in differentiated C2C12 myotubes as described before [25]. Data revealed that treatment with palmitate attenuated insulin-stimulated glucose uptake (Fig. 1a), the phosphorylation of IRS-1 at Ser 307 (Fig. 1b) and Ser 612 (Fig. 1c), and AKT at Thr 308 (Fig. 1d) and Thr 473 (Fig. 1e) in a dose-dependent manner in C2C12 myotubes.

**Fig. 2:**
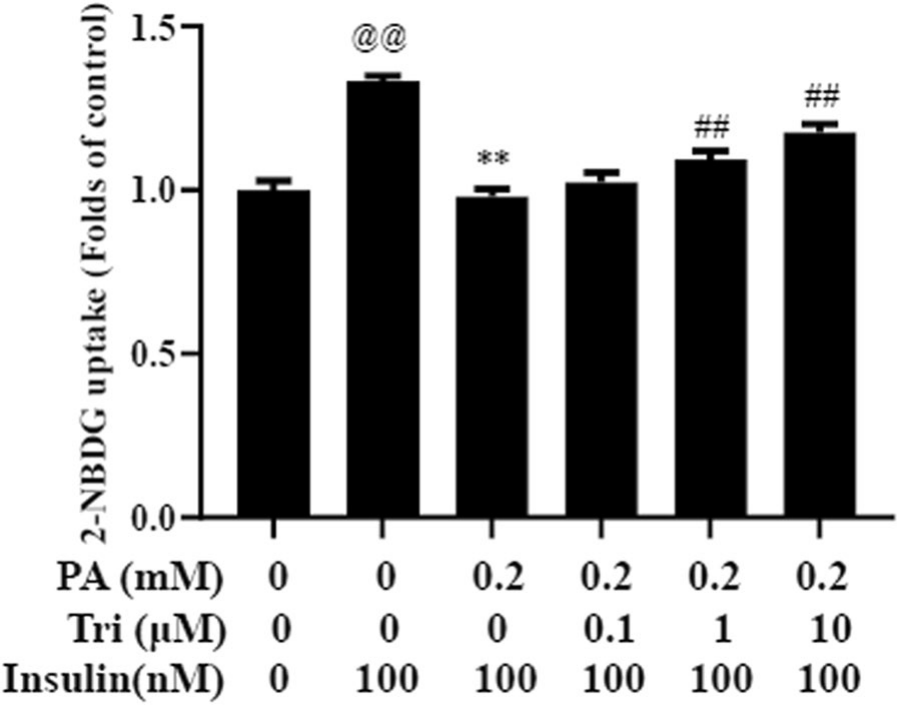
Trilobatin increased glucose uptake in palmitate-induced C2C12 myotubes. After C2C12 myotubes were incubated with 0.2 mM palmitate (PA) in the presence or absence of indicated concentrations of trilobatin (Tri) for 24 h, the cells were stimulated with 100 nM insulin for 30 min, and then incubated with 2-NBDG for 30 min. The uptake of 2-NBDG was determined as described in “Methods”. Data are mean ± SD (n = 6). ^@@^p < 0.01 vs. control (no insulin stimulation), **p < 0.01 vs. 100 nM insulin alone, ^##^p < 0.01 vs. 0.2 mM PA plus 100 nM insulin stimulation

To determine the potential impact of trilobatin on glucose uptake in insulin-resistant cells, the differentiated C2C12 myotubes were incubated with 0.2 mM palmitate in the presence or absence of 0, 0.1, 1.0 or 10 μM trilobatin for 24 h. After the cells were stimulated with 100 nM insulin for 30 min, 2-NBDG was added and incubated for another 30 min, and the glucose uptake was determined according to the suggestions from the supplier. The results demonstrated that treatment with palmitate induced a significant decrease of the uptake of 2-NBDG in C2C12 myotubes (p < 0.01), but incubation with trilobatin prevented that in a dose-dependent manner (p < 0.01) (Fig. 2).

### Trilobatin regulated the phosphorylation of IRS1 and Akt in palmitate-treated C2C12 myotubes

**Fig. 3:**
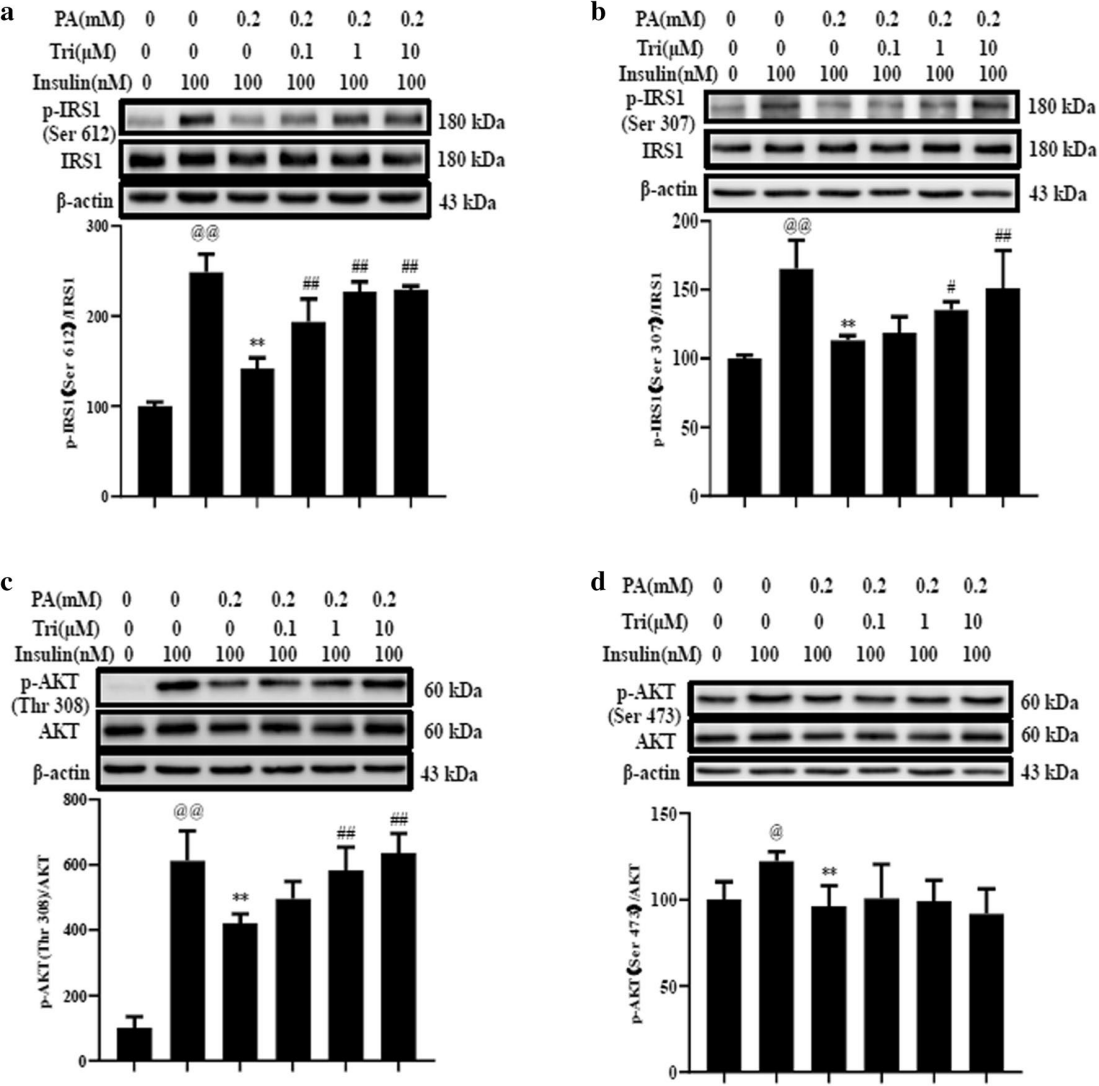
Trilobatin regulated the phosphorylation of IRS1 at Ser612 (**a**), Ser307 (**b**) and AKT at Thr 308 (**c**), Ser 473 (**d**) in palmitate-induced C2C12 myotubes. After C2C12 myotubes were incubated with 0.2 mM palmitate (PA) without or with indicated doses of trilobatin (Tri) for 24 h, the cells were washed once and stimulated with 100 nM insulin for 15 min. The cell lysates were used to determine the expression and phosphorylation of IRS1 at Ser 612 (**a**), Ser 307 (**b**) and AKT at Thr 308 (**c**), Ser 473 (**d**). Data are mean ± SD from three independent experiments. ^@@^p < 0.01 vs. control (no insulin stimulation), **p < 0.01 vs. 100 nM insulin alone, ^##^p < 0.01 vs. 0.2 mM PA plus 100 nM insulin stimulation

To observe the effect of trilobatin on insulin signaling molecules under the condition of insulin resistance, we determined the phosphorylation of insulin receptor substrate 1 (IRS1) and its downstream molecule AKT in palmitate-treated C2C12 myotubes, the results showed that trilobatin treatment recovered the phosphorylation of IRS1 at Ser 612 (Fig. 3a), Ser 307 (Fig. 3b) and AKT at Thr 308 (Fig. 3c) under stimulation with insulin, but there was no significant impact on the phosphorylation of AKT at Ser 473 (Fig. 3d).

### Trilobatin regulated the expression and distribution of GLUT4 in palmitate-treated C2C12 myotubes

**Fig. 4:**
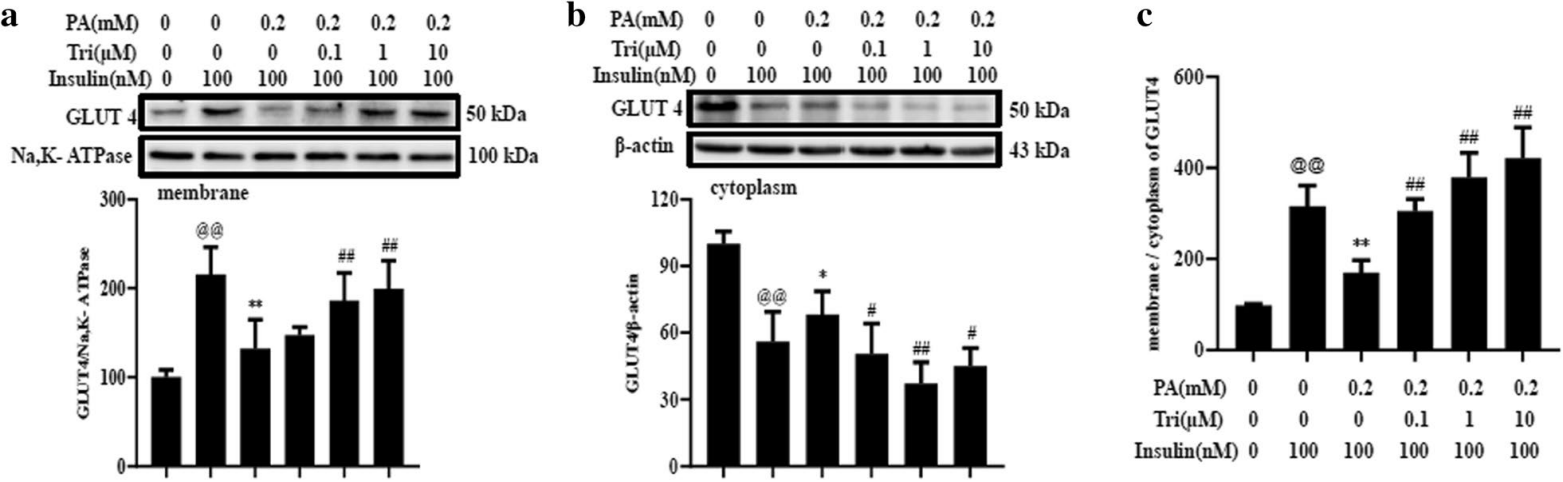
Trilobatin regulated the expression and translocation of GLUT4 in palmitate-treated C2C12 myotubes. After the differentiated C2C12 myotubes were incubated with 0.2 mM palmitate (PA) without or with indicated doses of trilobatin (Tri) for 24 h, the cells were washed once and stimulated with 100 nM insulin for 15 min. The cell lysates were used to determine the expression of GLUT4 in membranes (**a**) and cytoplasm (**b**), and the ratio of GLUT4 in membrane and cytoplasm was calculated (**c**). Data are mean ± SD from three independent experiments. ^@@^p < 0.01 vs. control (no insulin stimulation), *p < 0.05, **p < 0.01 vs. 100 nM insulin alone, ^##^p < 0.01 vs. 0.2 mM PA plus 100 nM insulin stimulation

To probe the actions of trilobatin on that in C2C12 myotubes challenged by palmitate, we determined the protein levels of GLUT4 in membrane and cytoplasm in palmitate-treated cells in the presence or absence of trilobatin. Data demonstrated that, compared with palmitate treatment alone, combination of palmitate and trilobatin increased the protein level of GLUT4 in the plasm membrane (Fig. 4a), but decreased the protein level of GLUT4 in the cytoplasm (Fig. 4b) in a dose-dependent manner. These results clearly indicated that treatment with trilobatin accelerated the translocation of GLUT4 from cytoplasm to plasma membrane. Consistence with this result, the ratio of membrane/cytoplasm of GLUT4 protein level was increased by trilobatin in a dose-dependent manner in palmitate-treated C2C12 myotubes (Fig. 4c).

### PI3K plays an essential role in trilobatin improving insulin resistance

**Fig. 5:**
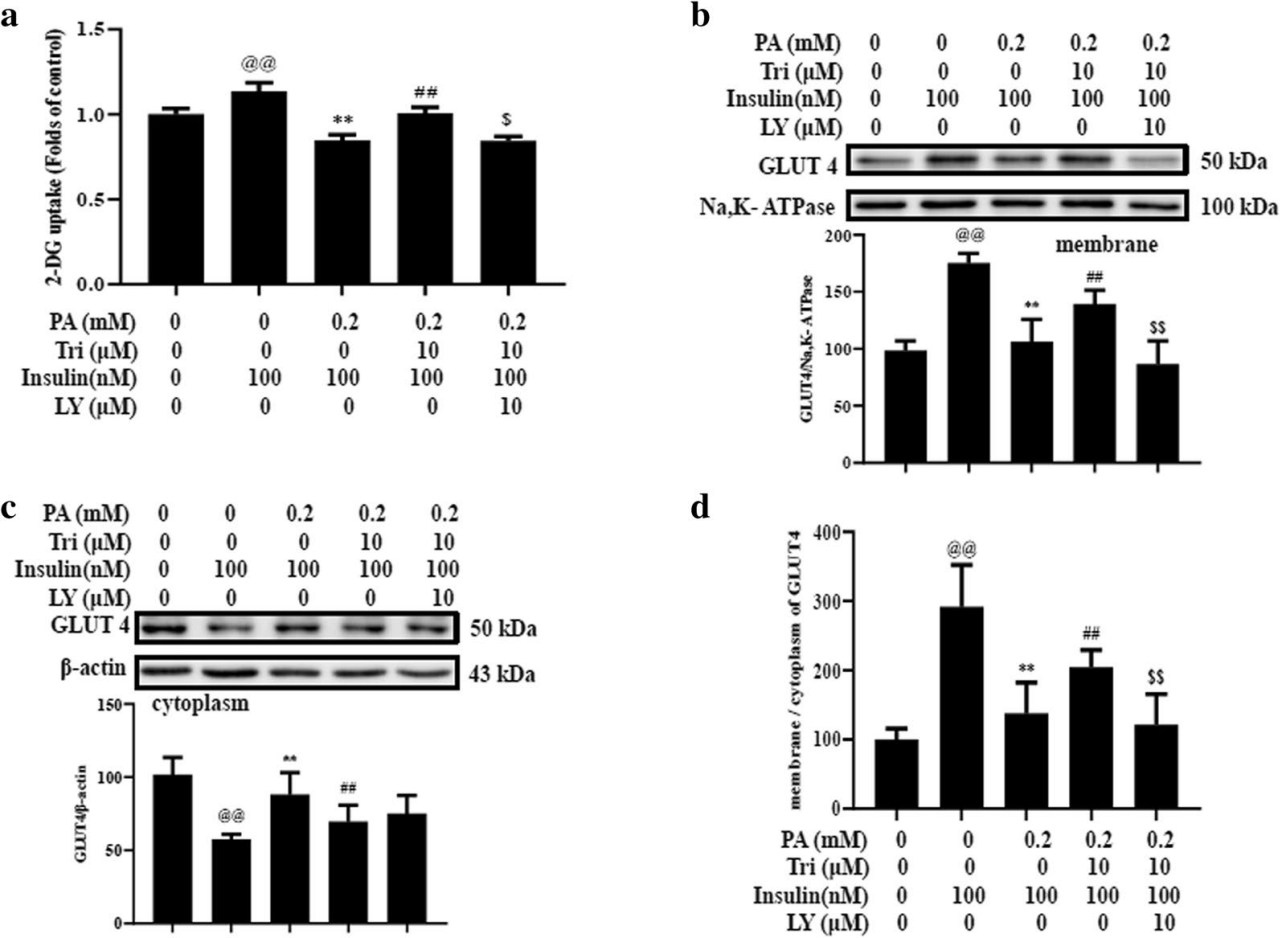
LY294002, an PI3K inhibitor, prevented the effects of trilobatin on glucose uptake and GLUT4 distribution in insulin-resistant C2C12 myotubes. After C2C12 myotubes were incubated with 0.2 mM palmitate (PA) without or with indicated doses of trilobatin (Tri) for 24 h, the cells were washed once and stimulated with 100 nM insulin for 30 min in the presence or absence of 10 μM LY294002 (LY), and then incubated with 2-NBDG for 30 min. **a** The uptake of 2-NBDG was determined as described in “Methods”. **b**, **c** the protein of GLUT4 in membrane and cytoplasm was detected with western blot. **d** The ratio of GLUT4 in membrane and cytoplasm was calculated. Data are mean ± SD from three independent experiments. ^@@^p < 0.01 vs. control (no insulin stimulation), **p < 0.01 vs. 100 nM insulin alone, ^##^p < 0.01 vs. 0.2 mM PA with 100 nM insulin stimulation, ^$^p < 0.05, ^$$^p < 0.01 vs. 0.2 mM PA combined with 10 μM Trilobatin and 100 nM insulin

To probe the cell signaling transduction, we determined the effect of pre-incubation with LY294002, a PI3K inhibitor, on the uptake of glucose in palmitate-treated C2C12 myotubes and the distribution of GLUT4 in plasma membrane and cytoplasm. The results revealed that pre-treatment with LY294002 prohibited the effects of trilobatin on glucose uptake (Fig. 5a), the distribution of GLUT4 (Fig. 5b–d) under insulin stimulation in insulin-resistant C2C12 myotubes.

### Trilobatin improved insulin resistance in ob/ob mice

**Fig. 6:**
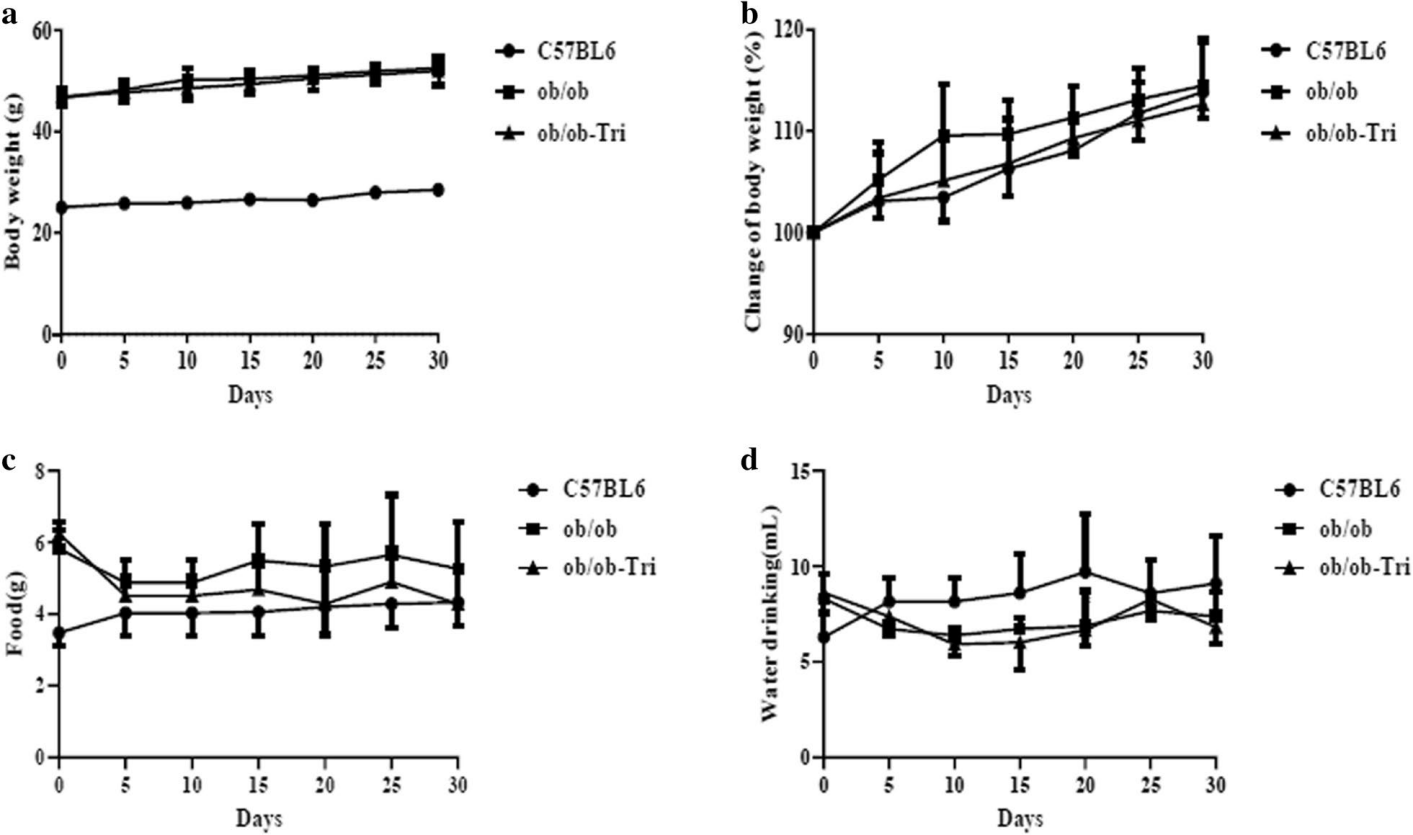
Effects of trilobatin on body weight, food and water drinking in ob/ob mice. Twenty male ob/ob mice were separated to two groups randomly, which were control (ob/ob) and trilobatin (ob/ob-Tri), ten same age of C57BL/6 male mice were used as negative control (C57BL6) in this study. During treatment with 10 mg/kg (intragastric, i.g.), the body weight (**a**), change of body weight (**b**), food (**c**) and water drinking (**d**) were monitored in ob/ob mice. Data are mean ± SD (n = 10)

The increasing evidence indicates that ob/ob mice are a reasonable experimental model of obesity-induced insulin resistance [26, 27]. In this study, we first observed the effects of trilobatin on body weight, food, and water drinking, data showed that treatment with 10 mg/kg trilobatin (i. g.) for 4 weeks had no significant impact on the body weight (Fig. 6a, F = 315.77), change of body weight (Fig. 6b, F = 0.538), food (Fig. 6c, F = 75.21) and water drinking (Fig. 6d, F = 6.612) in ob/ob mice.

**Fig. 7:**
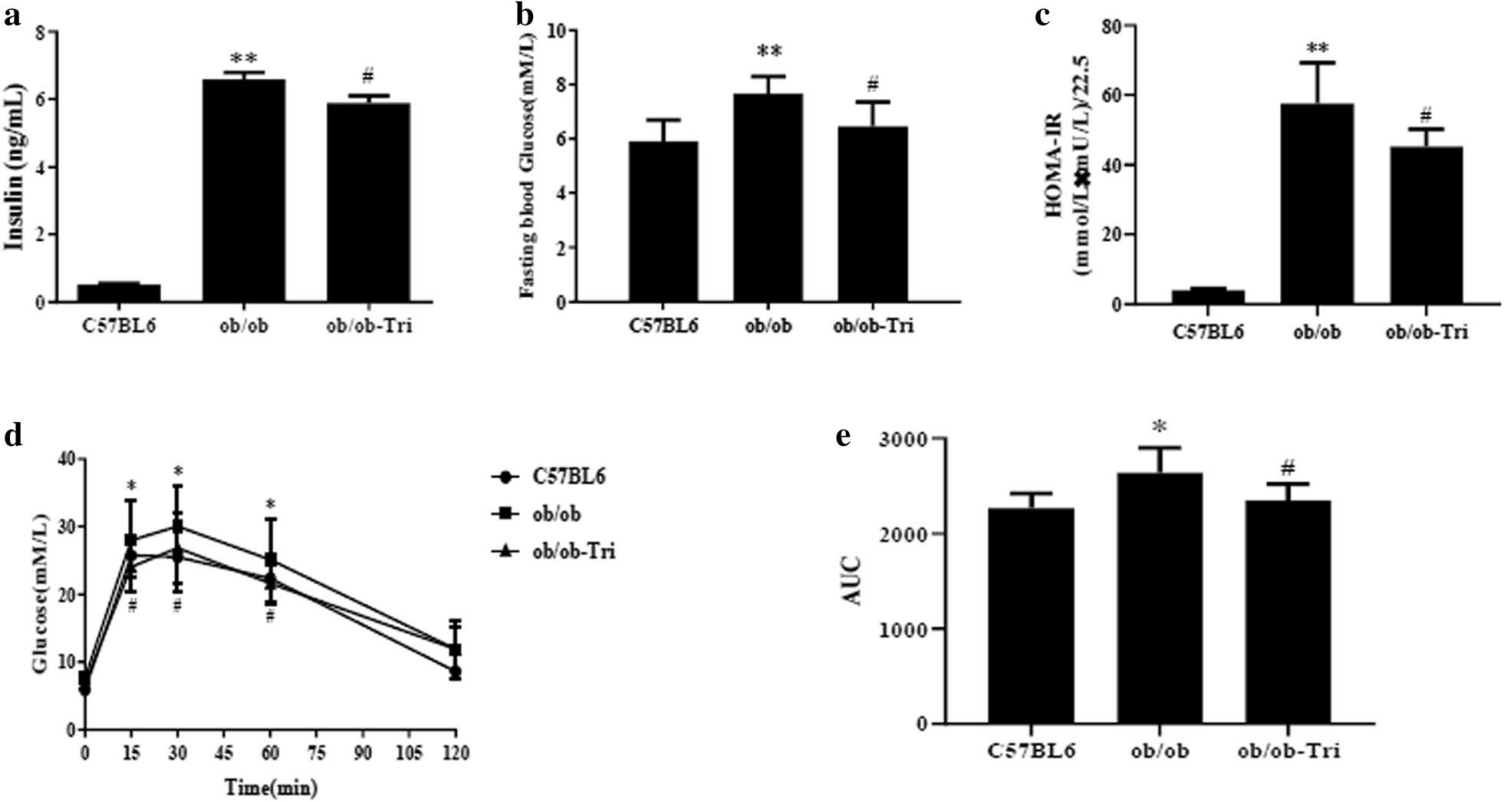
Trilobatin improved insulin resistance in ob/ob mice. Twenty male ob/ob mice were separated to two groups randomly, which were control (ob/ob) and trilobatin (ob/ob-Tri), ten same age of C57BL/6 male mice were used as negative control (C57BL6) in this study. After ob/ob mice were administrated with 10 mg/kg trilobatin (i. g.) for 4 weeks, the insulin content in serum (**a**), fasting blood glucose (**b**) and glucose tolerance test (**d**) was measured, HOMA-IR (**c**) and AUC (**e**) were calculated. Data are mean ± SD (n = 10). *p < 0.05, **p < 0.01 vs. C57BL6, ^#^p < 0.05 vs. ob/ob

At present, we also determined the influence of trilobatin on insulin resistance in ob/ob mice, the results demonstrated that treatment with 10 mg/kg trilobatin (intragastric, i.g.) for 4 weeks noticeably decreased fasting blood glucose (Fig. 7a) and insulin in serum (Fig. 7b), which was confirmed by the homeostasis model assessment of insulin resistance (HOMA-IR), a simple and efficient tool to evaluate the syndrome of insulin resistance (Fig. 7c). The results from the glucose tolerance test (GTT) showed that administration with trilobatin for 4 weeks significantly improved insulin resistance in ob/ob mice (Fig. 7d, e, p < 0.05, F = 0.695).

### Trilobatin regulated the phosphorylation of IRS1 and Akt in muscle tissue of ob/ob mice

**Fig. 8:**
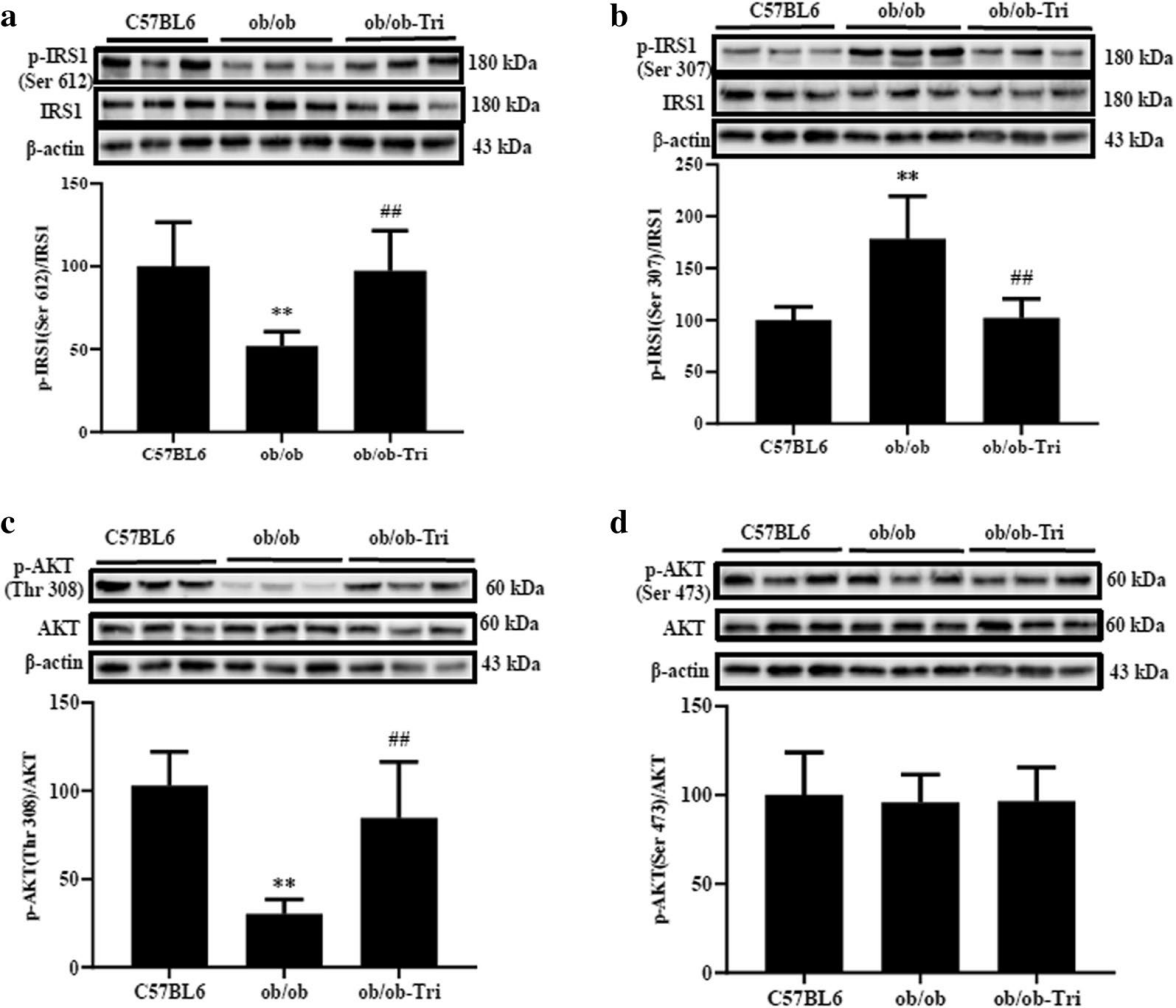
Trilobatin regulated the phosphorylation of IRS1 and AKT in muscle tissues of ob/ob mice. After the ob/ob mice were administrated with 10 mg/kg trilobatin (i. g.) for 4 weeks, the skeletal muscles were collected, and proteins were extracted, the expression of IRS1 and AKT, the phosphorylation of IRS1 at Ser 612 (**a**) and Ser 307 (**b**), and the phosphorylation AKT at Thr 308 (**c**) and Ser 473 (**d**) were detected with western blotting assay. Data are mean ± SD of randomly selected three from ten mice, and the experiments were repeated at least three times. **p < 0.01 vs. C57BL6, ^##^p < 0.01 vs. ob/ob

After ob/ob mice were administrated with 10 mg/kg trilobatin (i. g.) for 4 weeks, we found that trilobatin significantly improved the deleterious effect induced by obesity on the phosphorylation of IRS1 at Ser 612 (Fig. 8a) and Ser 307 (Fig. 8b), and Akt at Thr 308 (Fig. 8c), but treatment with 10 mg/kg trilobatin for 4 weeks had no effect on the phosphorylation of AKT at Ser 473 (Fig. 8d) in the skeletal muscle tissue of ob/ob mice.

### Trilobatin adjusted the expression and translocation of GLUT4 in the skeletal muscle of ob/ob mice

**Fig. 9:**
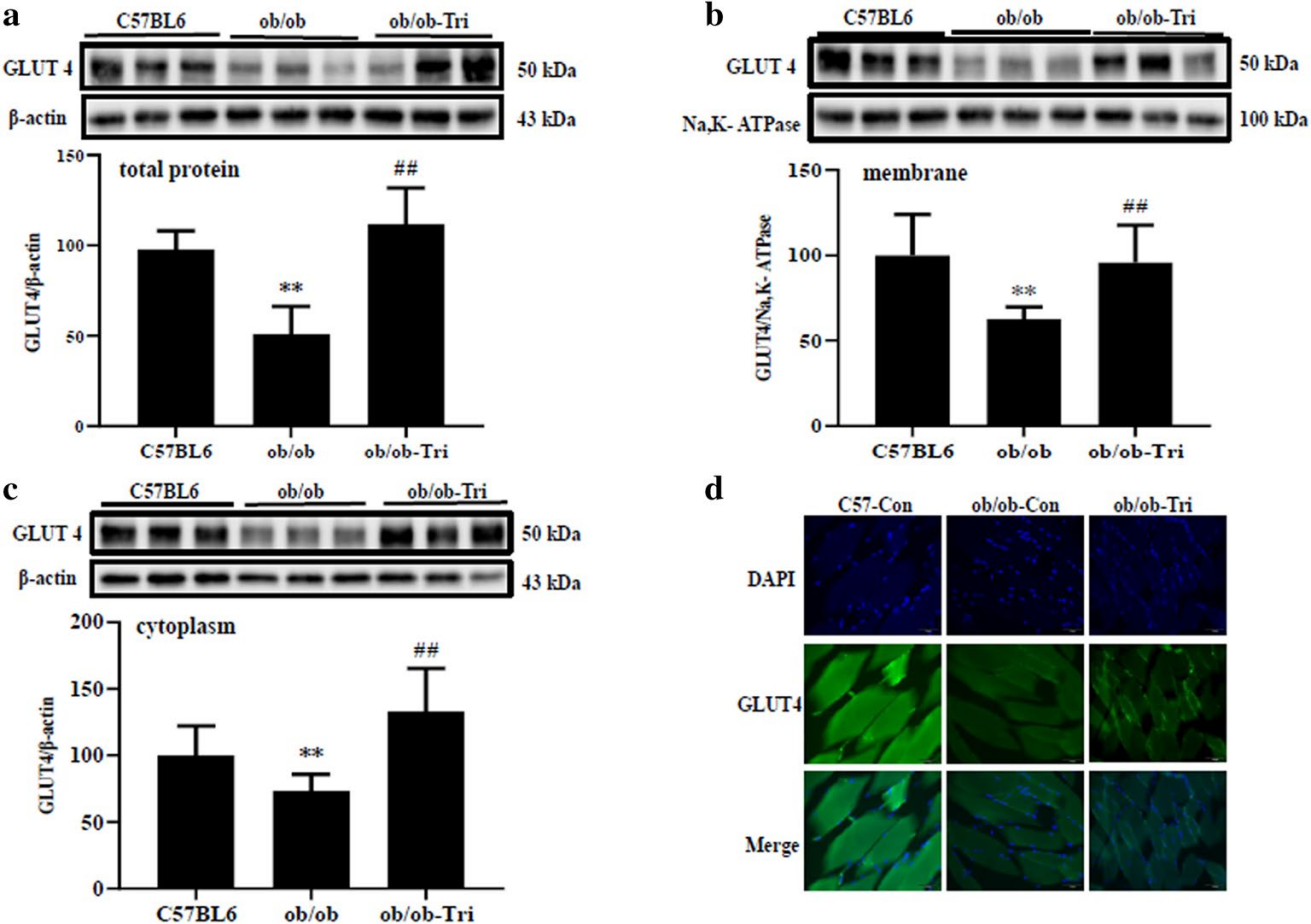
Trilobatin affected the expression and distribution of GLUT4 in muscle tissue of ob/ob mice. After the ob/ob mice were administrated with 10 mg/kg trilobatin (i. g.) for 4 weeks, the expression (**a**), distribution of GLUT 4 in membranes (**b**) and cytoplasm (**c**) were detected by western blot or immunofluorescence (**d**). Data are mean ± SD of randomly selected three from ten mice, and the experiments were repeated at least three times. **p < 0.01 vs. C57BL6, ^##^p < 0.01 vs. ob/ob

Insulin resistance induced a remarkably change in the expression and distribution of GLUT4 in skeletal muscle tissue [5, 28]. At present, after treated with 10 mg/kg trilobatin for 4 weeks, we determined the expression and translocation of GLUT4 in the skeletal muscle from ob/ob mice. The results showed that, compared to the same age of C57BL6 mice, the total protein of GLUT4 (Fig. 9a) was dramatically decreased, this phenomenon was also observed in plasma membrane (Fig. 9b) and cytoplasm (Fig. 9c). But after administrated with 10 mg/kg trilobatin (i. g.) for 4 weeks, the protein level of GLUT4 in the skeletal muscle tissue of ob/ob mice was significantly recovered (Fig. 9a–d).

## Discussion

It had been shown that IRS1, widely expressed in mammalian myocytes, was a direct physiological substrate of the insulin receptor and played an important role in insulin signal transmission, the phosphorylation of IRS1 on tyrosine residues could interact with phosphatidylinositol-3 kinase (PI3K), which phosphorylates phosphatidyl-inositol 2 phosphates (PIP2) into phosphatidyl-inositol 3 phosphates (PIP3), and recruited Akt to the plasma membrane through the plextrin homology (PH) domain. AKT then phosphorylated adenosine monophosphate-activated protein kinase (AMPK), an energy sensor in eukaryotic cells, which affected the translocation of GLUT4 to the plasma membrane [29, 30]. On the contrary, the dephosphorylation of IRS1 on tyrosine residues can potentially inactivate the whole process of insulin signal transduction, leading to insulin resistance [31].

Insulin resistance, a hallmark of obesity and type 2 diabetes, is characterized by the defect of insulin to stimulate glucose uptake and utilization in the liver, skeletal muscles and adipose tissues [1, 32]. It has become increasingly apparent that chronic elevation of plasma free fatty acids (FFAs) played an essential role in the impairment of insulin-stimulated glucose uptake in obesity and type 2 diabetes [33, 34]. Exposure of FFAs impaired glycogen synthesis and the transport and utilization of glucose in adipocytes, hepatocytes and skeletal muscle cells [35, 36]. Furthermore, prolonged exposure of FFAs induced the production of inflammatory factors and the activation of Jun N-terminal kinase (JNK), which accelerated β-oxidation of FFA, brought excessive electron flux in the mitochondrial respiratory chain, and subsequently caused the generation of reactive oxidant species (ROS) [37–39]. All these factors would damage the insulin signaling molecules by decreasing the phosphorylation of IRS and AKT, and impaired the expression and translocation of GLUT4, finally resulting in the defect of glucose uptake and utilization [40, 41]. Despite such data, the underlying mechanisms remain to be explored and the promising compounds are still limited. At present, we observed that incubation with palmitate-induced insulin resistance by attenuating insulin-stimulated glucose uptake, the phosphorylation of IRS1 and AKT, and the distribution of GLUT4, but trilobatin prohibited these effects of palmitate in C2C12 myotubes in a dose-dependent manner. The increasing data indicates that the PI3K/AKT signal pathway played a pivotal role on the uptake and utilization of glucose, and the full activation of AKT in response to insulin was major mediated by the phosphorylation at Thr 308 and Ser 473 [42, 43]. In the present study, treatment with trilobatin recovered the phosphorylation of AKT at Thr 308, and LY294002, an inhibitor of PI3K, blocked almost the effect of trilobatin on glucose uptake in palmitate-treated C2C12 myotubes. All these findings revealed that PI3K/AKT signaling pathway might be involved in trilobatin improving insulin resistance and glucose uptake in palmitate-treated C2C12 myotubes.

It has been widely reported that GLUT4, mainly expressed in skeletal muscle and white adipose tissue, plays an important role in insulin-induced glucose uptake [42, 44]. In these tissues, insulin induced the phosphorylation of IRS by binding to its tyrosine receptor. These phosphorylated proteins then activated PI3K by enhancing the phosphorylation of their downstream targets, including AKT and protein kinase C (PKC), which accelerated the translocation of GLUT4 from intracellular storage compartments to the plasma membrane [42, 43, 45]. In this study, our data demonstrated that consistence with the role on glucose uptake, incubation with trilobatin prevented the effects of palmitate on the translocation of GLUT4 from cytoplasm to plasma membrane in C2C12 myotubes, and in the presence of LY294002, the effect of trilobatin on the translocation of GLUT4 was inhibited evidently, suggested that AKT/GLUT4 signaling pathway was associated with trilobatin improving glucose uptake palmitate-treated C2C12 myotubes.

To explore the effects of trilobatin on insulin resistance in vivo, we determined the role of trilobatin in ob/ob mice, an interesting genetically obese mouse model characterized by hyperglycemia, hyperinsulinemia, and insulin resistance. The results demonstrated that administration with trilobatin for 4 weeks significantly decreased the contents of fasting blood glucose and insulin, improved insulin resistance, increased the phosphorylation of IRS1 at Ser 612, AKT at Thr 308 and decreased the phosphorylation of IRS1 at Ser 307, and recovered the expression and translocation of GLUT4 in ob/ob mice. All these findings indicated that trilobatin might be a benefit to alleviate insulin resistance induced by obesity, and a promising compound for the treatment of obesity and type 2 diabetes.

## Conclusion

There is now a wealth of evidence that IRS-AKT-GLUT4 signal pathway plays an important role in the development of insulin resistance in obese animal models [44, 46]. In the present study, we demonstrated that administration with trilobatin alleviated obesity-induced insulin resistance by decreasing the levels of fasting blood glucose and insulin, improving glucose tolerance, and these beneficial effects were associated with trilobatin ameliorating the defects of IRS1 and AKT phosphorylation, recovering the translocation of GLUT4 in ob/ob mice. The results of the current study strongly support the concept that the IRS-AKT-GLUT4 signaling pathway is involved in trilobatin improving insulin resistance in C2C12 myotubes and ob/ob mice.

## Supplementary information

**Additional file 1.** The differentiation of C2C12 myotubes. After C2C12 myoblasts were incubated with differentiated media as described in Methods. The myotubes were observed (A), and the protein markers MYH1 (B) and MyoD1 (C) were determined with western blot. Data are mean ± SD from three independent experiments. *, p < 0.05, **, p < 0.01 vs. control (0 day).

## Data Availability

The datasets used and/or analyzed during the current study are available from the corresponding author on reasonable request.

## Abbreviations

BSA: Bovine serum albumins
GLUT4: Glucose transporter 4
GTT: Glucose tolerance test
HFD: High-fat diet
IR: Insulin resistance
2-NBDG: 2-(*N*-(7-Nitrobenz-2-oxa-1,3-diazol-4-yl) amino)-2-deoxyglucose
PKB: Protein kinase B
PKC: Protein kinase C
STZ: Streptozotocin

## References

[1] Al-Sulaiti H, Diboun I, Agha MV, Mohamed FFS, Atkin S, Domling AS (2019). Metabolic signature of obesity-associated insulin resistance and type 2 diabetes. J Transl Med.

[2] Hagman E, Besor O, Hershkop K, Santoro N, Pierpont B, Mata M (2019). Relation of the degree of obesity in childhood to adipose tissue insulin resistance. Acta Diabetol.

[3] DeFronzo RA, Gunnarsson R, Bjorkman O, Olsson M, Wahren J (1985). Effects of insulin on peripheral and splanchnic glucose metabolism in noninsulin-dependent (type II) diabetes mellitus. J Clin Invest.

[4] Thiebaud D, Jacot E, DeFronzo RA, Maeder E, Jequier E, Felber JP (1982). The effect of graded doses of insulin on total glucose uptake, glucose oxidation, and glucose storage in man. Diabetes.

[5] Fujiwara Y, Tsukahara C, Ikeda N, Sone Y, Ishikawa T, Ichi I (2017). Oleuropein improves insulin resistance in skeletal muscle by promoting the translocation of GLUT4. J Clin Biochem Nutr.

[6] Li Z, Zhu Y, Li C, Tang Y, Jiang Z, Yang M (2018). Liraglutide ameliorates palmitate-induced insulin resistance through inhibiting the IRS-1 serine phosphorylation in mouse skeletal muscle cells. J Endocrinol Invest.

[7] Sanvee GM, Panajatovic MV, Bouitbir J, Krahenbuhl S (2019). Mechanisms of insulin resistance by simvastatin in C2C12 myotubes and in mouse skeletal muscle. Biochem Pharmacol.

[8] Xiao Z, Zhang Y, Chen X, Wang Y, Chen W, Xu Q (2017). Extraction, identification, and antioxidant and anticancer tests of seven dihydrochalcones from Malus 'Red Splendor' fruit. Food Chem.

[9] Duge de Bernonville T, Guyot S, Paulin JP, Gaucher M, Loufrani L, Henrion D (2010). Dihydrochalcones: implication in resistance to oxidative stress and bioactivities against advanced glycation end-products and vasoconstriction. Phytochemistry.

[10] Yang WM, Liu JK, Qin XD, Wu WL, Chen ZH (2004). Antioxidant activities of three dihydrochalcone glucosides from leaves of *Lithocarpus pachyphyllus*. Z Naturforsch C J Biosci.

[11] Fan X, Zhang Y, Dong H, Wang B, Ji H, Liu X (2015). Trilobatin attenuates the LPS-mediated inflammatory response by suppressing the NF-kappaB signaling pathway. Food Chem.

[12] Yin S, Zhang X, Lai F, Liang T, Wen J, Lin W (2018). Trilobatin as an HIV-1 entry inhibitor targeting the HIV-1 Gp41 envelope. FEBS Lett.

[13] Wang J, Huang Y, Li K, Chen Y, Vanegas D, McLamore ES (2016). Leaf extract from *Lithocarpus polystachyus* Rehd. Promote glycogen synthesis in T2DM mice. PLoS ONE.

[14] Katsuda Y, Sasase T, Tadaki H, Mera Y, Motohashi Y, Kemmochi Y (2015). Contribution of hyperglycemia on diabetic complications in obese type 2 diabetic SDT fatty rats: effects of SGLT inhibitor phlorizin. Exp Anim.

[15] Wu CH, Ho YS, Tsai CY, Wang YJ, Tseng H, Wei PL (2009). In vitro and in vivo study of phloretin-induced apoptosis in human liver cancer cells involving inhibition of type II glucose transporter. Int J Cancer.

[16] Figueiredo-Gonzalez M, Grosso C, Valentao P, Andrade PB (2016). alpha-Glucosidase and alpha-amylase inhibitors from *Myrcia* spp.: a stronger alternative to acarbose?. J Pharm Biomed Anal.

[17] Uto H (2009). alpha-Glucosidase inhibitor acarbose and sequestome 1/A170/p62 deficient mice: a promising therapy and unique model for non-alcoholic fatty liver disease. Hepatol Res.

[18] Guo M, Li Y, Wang Y, Li Z, Li X, Zhao P (2019). eEF1A2 exacerbated insulin resistance in male skeletal muscle via PKCbeta and ER stress. J Endocrinol.

[19] Shen S, Liao Q, Zhang T, Pan R, Lin L (2019). Myricanol modulates skeletal muscle-adipose tissue crosstalk to alleviate high-fat diet-induced obesity and insulin resistance. Br J Pharmacol.

[20] Lee YS, Cha BY, Saito K, Yamakawa H, Choi SS, Yamaguchi K (2010). Nobiletin improves hyperglycemia and insulin resistance in obese diabetic ob/ob mice. Biochem Pharmacol.

[21] Liu J, Yin F, Xiao H, Guo L, Gao X (2012). Glucagon-like peptide 1 receptor plays an essential role in geniposide attenuating lipotoxicity-induced beta-cell apoptosis. Toxicol In Vitro.

[22] Chen Y, Zhang J, Zhang XY (2015). 2-NBDG as a marker for detecting glucose uptake in reactive astrocytes exposed to oxygen-glucose deprivation in vitro. J Mol Neurosci.

[23] Zou C, Wang Y, Shen Z (2005). 2-NBDG as a fluorescent indicator for direct glucose uptake measurement. J Biochem Biophys Methods.

[24] Lluis F, Perdiguero E, Nebreda AR, Munoz-Canoves P (2006). Regulation of skeletal muscle gene expression by p38 MAP kinases. Trends Cell Biol.

[25] Nieuwoudt S, Mulya A, Fealy CE, Martelli E, Dasarathy S, Naga Prasad SV (2017). In vitro contraction protects against palmitate-induced insulin resistance in C2C12 myotubes. Am J Physiol Cell Physiol.

[26] Belfiore F, Rabuazzo AM, Iannello S, Vasta D, Campione R (1984). Insulin resistance in the obese hyperglycemic (ob/ob) mouse. Failure of hyperinsulinemia to activate hepatic pyruvate kinase (PK). Metabolism.

[27] Clarke PV, Kissebah AH, Hope-Gill H, Vydelingum N, Tulloch B, Fraser TR (1975). The role of calcium in insulin action. IV. Mechanism of insulin resistance in adipose tissue of obese (ob/ob) mice and old Wistar rats. Eur J Clin Invest.

[28] Fang P, Yu M, Zhang L, Wan D, Shi M, Zhu Y (2017). Baicalin against obesity and insulin resistance through activation of AKT/AS160/GLUT4 pathway. Mol Cell Endocrinol.

[29] Lima MH, Ueno M, Thirone AC, Rocha EM, Carvalho CR, Saad MJ (2002). Regulation of IRS-1/SHP2 interaction and AKT phosphorylation in animal models of insulin resistance. Endocrine.

[30] Ren L, Zhou X, Huang X, Wang C, Li Y (2019). The IRS/PI3K/Akt signaling pathway mediates olanzapine-induced hepatic insulin resistance in male rats. Life Sci.

[31] Babu S, Krishnan M, Rajagopal P, Periyasamy V, Veeraraghavan V, Govindan R (2020). Beta-sitosterol attenuates insulin resistance in adipose tissue via IRS-1/Akt mediated insulin signaling in high fat diet and sucrose induced type-2 diabetic rats. Eur J Pharmacol.

[32] Smith U, Axelsen M, Carvalho E, Eliasson B, Jansson PA, Wesslau C (1999). Insulin signaling and action in fat cells: associations with insulin resistance and type 2 diabetes. Ann N Y Acad Sci.

[33] Boden G (2003). Effects of free fatty acids (FFA) on glucose metabolism: significance for insulin resistance and type 2 diabetes. Exp Clin Endocrinol Diabetes.

[34] Christiansen E, Urban C, Merten N, Liebscher K, Karlsen KK, Hamacher A (2008). Discovery of potent and selective agonists for the free fatty acid receptor 1 (FFA(1)/GPR40), a potential target for the treatment of type II diabetes. J Med Chem.

[35] Chavez JA, Summers SA (2003). Characterizing the effects of saturated fatty acids on insulin signaling and ceramide and diacylglycerol accumulation in 3T3-L1 adipocytes and C2C12 myotubes. Arch Biochem Biophys.

[36] Hirabara SM, Curi R, Maechler P (2010). Saturated fatty acid-induced insulin resistance is associated with mitochondrial dysfunction in skeletal muscle cells. J Cell Physiol.

[37] Yuzefovych L, Wilson G, Rachek L (2010). Different effects of oleate vs. palmitate on mitochondrial function, apoptosis, and insulin signaling in L6 skeletal muscle cells: role of oxidative stress. Am J Physiol Endocrinol Metab.

[38] Nakamura S, Takamura T, Matsuzawa-Nagata N, Takayama H, Misu H, Noda H (2009). Palmitate induces insulin resistance in H4IIEC3 hepatocytes through reactive oxygen species produced by mitochondria. J Biol Chem.

[39] Gao D, Nong S, Huang X, Lu Y, Zhao H, Lin Y (2010). The effects of palmitate on hepatic insulin resistance are mediated by NADPH Oxidase 3-derived reactive oxygen species through JNK and p38MAPK pathways. J Biol Chem.

[40] Kadotani A, Tsuchiya Y, Hatakeyama H, Katagiri H, Kanzaki M (2009). Different impacts of saturated and unsaturated free fatty acids on COX-2 expression in C(2)C(12) myotubes. Am J Physiol Endocrinol Metab.

[41] Zhang Y, Yang S, Zhang M, Wang Z, He X, Hou Y (2019). Glycyrrhetinic acid improves insulin-response pathway by regulating the balance between the Ras/MAPK and PI3K/Akt pathways. Nutrients.

[42] Ducluzeau PH, Fletcher LM, Welsh GI, Tavare JM (2002). Functional consequence of targeting protein kinase B/Akt to GLUT4 vesicles. J Cell Sci.

[43] Hill MM, Clark SF, Tucker DF, Birnbaum MJ, James DE, Macaulay SL (1999). A role for protein kinase Bbeta/Akt2 in insulin-stimulated GLUT4 translocation in adipocytes. Mol Cell Biol.

[44] Li W, Liang X, Zeng Z, Yu K, Zhan S, Su Q (2016). Simvastatin inhibits glucose uptake activity and GLUT4 translocation through suppression of the IR/IRS-1/Akt signaling in C2C12 myotubes. Biomed Pharmacother.

[45] Egawa K, Maegawa H, Shi K, Nakamura T, Obata T, Yoshizaki T (2002). Membrane localization of 3-phosphoinositide-dependent protein kinase-1 stimulates activities of Akt and atypical protein kinase C but does not stimulate glucose transport and glycogen synthesis in 3T3-L1 adipocytes. J Biol Chem.

[46] Sharma BR, Kim HJ, Rhyu DY (2015). Caulerpa lentillifera extract ameliorates insulin resistance and regulates glucose metabolism in C57BL/KsJ-db/db mice via PI3K/AKT signaling pathway in myocytes. J Transl Med.

