# Supplementary figures and images for: Trilobatin ameliorates insulin resistance through IRS-AKT-GLUT4 signaling pathway in C2C12 myotubes and ob/ob mice
Source: Chin Med. 2020 Oct 12;15:110. doi: 10.1186/s13020-020-00390-2 (PMC7552530; doi:10.1186/s13020-020-00390-2)

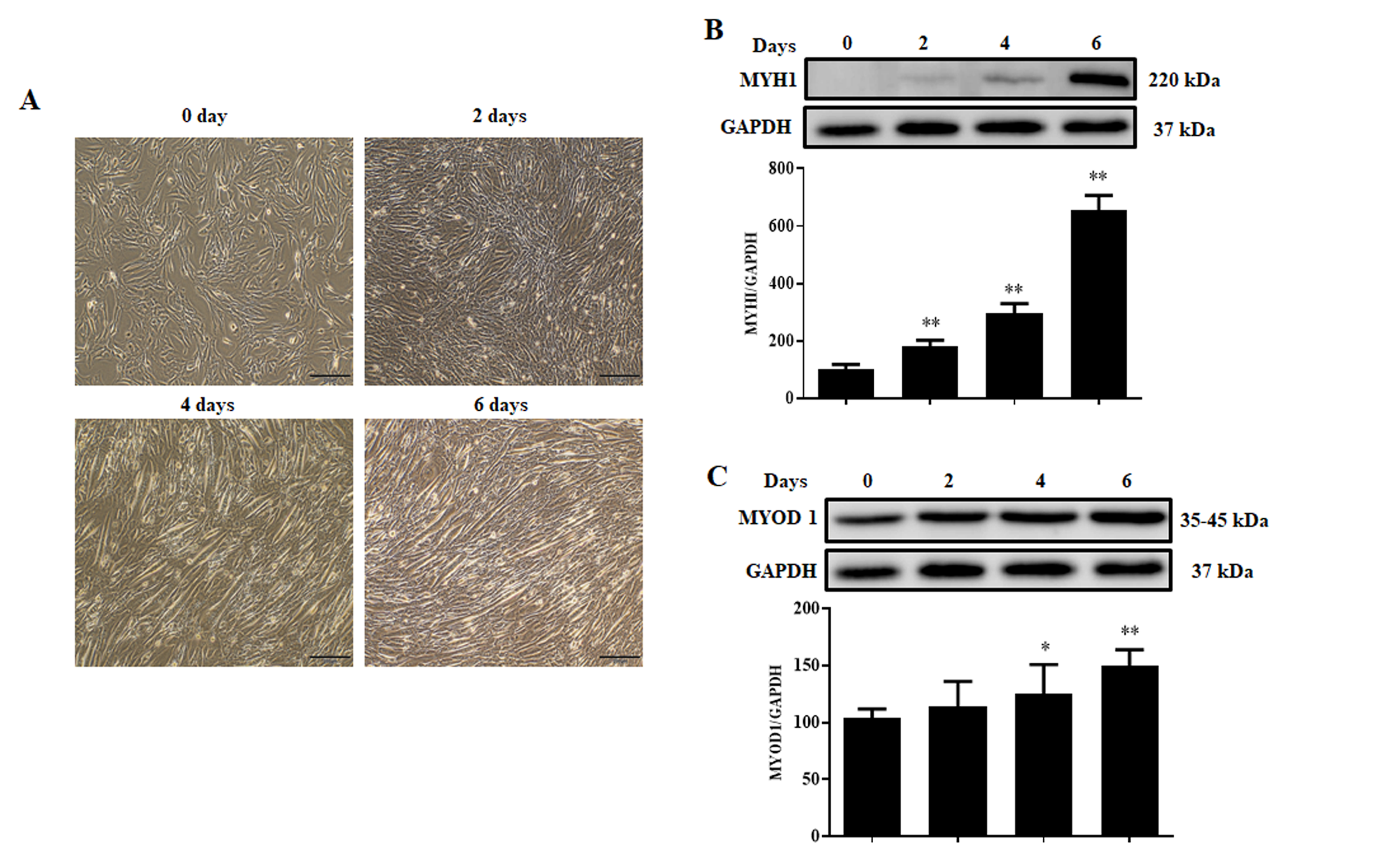

Supplement: Supplementary file 1 — Additional file 1. The differentiation of C2C12 myotubes. After C2C12 myoblasts were incubated with differentiated media as described in Methods. The myotubes were observed (A), and the protein markers MYH1 (B) and MyoD1 (C) were determined with western blot. Data are mean ± SD from three independent experiments. *, p < 0.05, **, p < 0.01 vs. control (0 day). [file 13020_2020_390_MOESM1_ESM.tif]
